# Emergency Surgery for Acute Left-Sided Complicated Diverticulitis in the Elderly: What Are the Predictor Factors of Mortality and Morbidity?

**DOI:** 10.3390/jcm14176298

**Published:** 2025-09-06

**Authors:** Samuele Vaccari, Basilio Pirrera, Alessandro Ussia, Augusto Lauro, Margherita Minghetti, Maurizio Cervellera, Vito D’Andrea, Valeria Tonini

**Affiliations:** 1 Unit of General and Emergency Surgery-Bentivoglio, Surgical Department-AUSL Bologna, 40010 Bologna, Italy; 2 Unit of General and Emergency Surgery-Infermi Hospital, Rimini-AUSL Romagna, 47921 Rimini, Italy; eliopirrera@virgilio.it; 3 Department of Surgery, Sapienza University of Rome, 00161 Rome, Italy; augusto.lauro@uniroma1.it (A.L.); vito.dandrea@uniroma1.it (V.D.); 4 Department of Medical and Surgical Sciences, University of Bologna, 40138 Bologna, Italy; margherita.minghetti@studio.unibo.it (M.M.); valeria.tonini@unibo.it (V.T.); 5 Department of General Surgery, Ospedale Santissima Annunziata, 74121 Taranto, Italy; maurizio.cervellera@asl.taranto.it

**Keywords:** colorectal surgery, elderly, diverticulitis, emergency surgery

## Abstract

**Introduction:** Diverticular disease is common in Western countries, and the frequency of emergency operations for acute left-sided complicated diverticulitis (ALCD) has increased over the past 15 years. **Methods:** A total of 49 patients aged over 80 years and 125 younger patients who underwent emergency surgery for ALCD between October 2018 and June 2025 were analyzed. Demographics and postoperative outcomes were compared between the groups. Multivariate logistic regression was used to assess the association between age and postoperative morbidity and mortality. A separate regression model was used to identify risk factors for postoperative mortality and morbidity, specifically in elderly patients. **Results:** Significant differences between the two groups were found in sex distribution (*p* < 0.001), cardiovascular comorbidities (*p* < 0.001), chronic renal insufficiency (CRI) (*p* < 0.001), ASA score (*p* < 0.001), ALCD severity according to the modified Hinchey classification (*p* = 0.006), Mannheim Peritonitis Index (MPI) (*p* = 0.021), postoperative complications (*p* < 0.001), and 90-day mortality rates (*p* < 0.001). Advanced age was a significant predictor of 90-day postoperative mortality and morbidity. In the elderly subgroup, an ASA score ≥ 3, MPI > 25, CRI, and COPD were identified as independent predictors of 90-day postoperative mortality and morbidity. **Conclusions:** Advanced age is an independent risk factor for 90-day postoperative mortality and morbidity following emergency surgery for ALCD. In patients over 80 years, an ASA score ≥ 3, CRI, COPD, and MPI ≥ 25 are associated with a poorer prognosis.

## 1. Introduction

Diverticular disease is common in Western countries, with an overall prevalence of approximately 27%. Recent data suggest that up to 50% of individuals older than 60 years of age have colonic diverticula, with 10% to 25% of these individuals developing complications such as diverticulitis. Hospitalizations for diverticular disease have also increased in recent years [1]. One American study examining hospitalization rates between 1998 and 2005 [2] reported a 26% rise in admissions for diverticular disease over the eight-year study period. Similar trends have been observed in Canadian and European data during the same timeframe.

The progressive aging of the population presents a growing challenge for surgeons and other healthcare providers, particularly in managing elderly patients who require emergency surgical interventions for complicated acute diverticulitis [3,4]. Increased postoperative mortality among elderly surgical patients is well documented in the literature [5,6,7,8,9,10,11,12,13,14,15,16,17].

The aim of this study was to evaluate the impact of advanced age (≥80) on treatment and surgical outcomes in patients undergoing colonic resections for ALCD, and identify independent factors associated with postoperative mortality and morbidity in this older population.

## 2. Materials and Methods

The study was designed as a retrospective case–control, observational study. Between October 2018 and June 2025, we evaluated 174 consecutive surgical procedures performed for ACLD at “St Orsola Hospital—Emergency Surgery Unit—University of Bologna” and “Ospedale di Bentivoglio/AUSL-Bologna—Department of Surgical Sciences”, sharing data in a common prospective database started in 2017. The study received approval from the hospital’s institutional review board (156/2018/Oss/AOUBo, 18/04/18). Informed consent was obtained from every participant.

Relevant clinical data were retrospectively extracted from hospital electronic records and patient charts. The following variables were collected and analyzed: Demographics (including Age and Sex), Comorbidities (including Cardio-Vascular, Diabetes, COPD, CRI and Immunosuppression), Intraoperative Variables (including Operative procedure and Duration of procedure) and Postoperative Outcomes (Length of hospital stay, 90-day mortality and 30-day Readmission). Patients with known immune diseases or taking high-dose steroids and/or other immunosuppressants for diseases such as rheumatological disorders and inflammatory bowel disease were defined as immunosuppressed. Standard blood tests including WBC and CRP and abdominal CT scans were obtained for all patients before admission to the surgical department. We used the Hinchey classification to determine the severity of complicated diverticulitis. Experienced surgeons or surgical residents under their direct supervision performed all surgical procedures. The patients’ general well-being was assessed based on the American Society of Anesthesiologists (ASA) score.

Postoperative complications were classified according to the Clavien–Dindo classification [18]. Grade I includes minor complications that do not require any intervention and that can be treated with routine medications like antipyretics, analgesics, diuretics, or physiotherapy. Grade II includes conditions that require major pharmacological intervention, like respiratory infections, ascites, blood transfusions, and asymptomatic pulmonary embolism. Grade III includes any complication requiring a surgical, endoscopic, or radiological intervention, like a respiratory infection requiring bronchoscopy, a pleural effusion requiring drainage, ascites or an abdominal collection requiring percutaneous drainage, and reoperation for abdominal collection, bleeding, or other reasons. Grade IV includes patients with life-threatening complications requiring admission to the Intensive Care Unit, and Grade V includes death in the postoperative period. Postoperative mortality was assessed over a 90-day period. Analyses were performed for the entire study population and separately for the elderly group.

To assess the impact of age on postoperative outcomes, patients were divided into two groups: Group 1 (n = 125), patients younger than 80 years; Group 2 (n = 49), patients aged 80 years or older.

The primary objective of the study was to determine whether advanced age was associated with increased postoperative mortality and morbidity following surgery for ALCD. The secondary objective was to identify independent predictive factors of postoperative mortality and morbidity in the elderly cohort. ALCD severity was classified according to the modified Hinchey classification [19]. The severity of abdominal sepsis was assessed using the Mannheim Peritonitis Index (MPI), with a score > 25 considered indicative of severe sepsis [20,21,22], as shown in Table 1.

Data were entered into a Microsoft Access database (Microsoft Corp., Redmond, WA, USA), and statistical analysis was conducted using SPSS version 10.0 (SPSS Inc., Chicago, IL, USA). Categorical variables were compared using the Pearson chi-squared test. Continuous variables were analyzed using Student’s *t*-test.

A backward stepwise logistic regression analysis was performed to identify covariates associated with postoperative morbidity and mortality. The inclusion threshold for candidate variables was set at *p* < 0.15, as recommended by Bursac et al. [23]. The author describes in his article how the small sample size limits the statistical power of the analysis, and a higher *p*-value may increase the chances of detecting potentially important, even if minor, effects. Statistical significance was defined as *p* ≤ 0.05. Results are reported as odds ratios (ORs) with 95% confidence intervals (CIs).

## 3. Results

Patients were divided into two groups based on age: Group 1 (n = 125), patients younger than 80 years; Group 2 (n = 49), patients aged 80 years or older. As shown in Table 2 the two groups differed significantly in sex distribution (*p* < 0.001), presence of cardiovascular comorbidities (*p* < 0.001), chronic renal insufficiency (CRI) (*p* < 0.001), ASA score (*p* < 0.001), ALCD severity according to the modified Hinchey classification (*p* = 0.006), and severity of peritonitis according to the MPI (*p* = 0.021).

In particular, the cohort of patients had a mean age at surgery of 72.1 ± 12.2 years; male patients were more frequent in Group 1 (57.6%). As previously written, the overall comorbidity rate differed between the two groups: in the older group, about 90% of patients had a cardiovascular risk factor compared to 45% of patients in the other group (*p* < 0.01). CRI was more frequent in Group 2 with a statistical difference (*p* < 0.01). Diabetes, COPD, and Immunosuppression did not differ significantly between the two groups. Not surprisingly, the ASA classification also differed significantly (*p* < 0.01) between the groups. Older patients presented a higher Mannheim Peritonitis Index than younger ones (*p* = 0.021).

Table 3 summarizes the intraoperative and post-operative characteristics of the study cohorts. The overall median operative time was 135.7 ± 2.1 min. There was a trend toward longer duration of surgeries in older patients; in particular, median operative time was 135.7 ± 38.6 min in Group 1 and 136.6 ± 48.6 min in Group 2, without significant differences between the two groups (*p* = 0.898). Eleven patients were treated with laparoscopic lavage and 163 patients underwent an open approach. There were no differences in the type of surgical procedures performed between the two groups. However, Group 2 experienced a significantly higher rate of postoperative complications (*p* < 0.001) and 90-day mortality (*p* < 0.001). There were 51 minor complications (including 20 wound infections, 25 respiratory infections, 6 blood transfusions) and 15 major complications (including 10 abdominal collections requiring percutaneous drainage, 3 reoperations for abdominal collection or bleeding and 2 life-threatening complications requiring admission to the Intensive Care Unit without reoperation). All reported patient deaths occurred due to septic shock; five patients died after re-intervention. However, no significant differences were recorded between the two groups. The overall median hospital stay was 13.81 ± 13.3 days, one day longer in the older group (*p* = ns).

After univariate analysis between the two groups, we performed a backward stepwise logistic regression analysis to identify covariates associated with postoperative morbidity and mortality. Logistic regression analysis identified several variables (Table 4 and Table 5) as significant predictive factors for postoperative morbidity and 90-day mortality following surgery for ALCD. Age ≥ 80 years emerged as an independent risk factor for adverse postoperative outcomes and increased 90-day mortality. Patients aged >80 years had an almost threefold increased risk of developing an adverse outcome and had a twofold increased risk of 90-day mortality. Also, cardiovascular comorbidities were an independent risk factor for adverse outcomes and CRI, an ASA score, or Mannheim Peritonitis Index > 25 were independent risk factors for both postoperative outcome and 90-day mortality.

To further explore predictive factors in elderly patients, a subgroup analysis was conducted on octogenarians. As shown in Table 6 and Table 7, factors significantly associated with poor surgical outcomes in this group included cardiovascular comorbidities (*p* = 0.027), chronic obstructive pulmonary disease (COPD) (*p* = 0.016), chronic renal insufficiency (*p* = 0.025 for morbidity and *p* = 0.031 for mortality), and a high ASA score (*p* = 0.004), and MPI > 25 (*p* < 0.001).

Logistic regression analysis in the elderly subgroup confirmed these variables (Table 8) as independent predictive factors for postoperative morbidity and 90-day mortality following surgery for ALCD.

## 4. Discussion

Life expectancy is steadily increasing in Western countries, and the frequency of emergency operations for acute left-sided complicated diverticulitis (ALCD) has also risen over the past 15 years in both Western and Asian populations [19,20,24]. This study demonstrates that advanced age (≥80 years) is independently associated with increased postoperative morbidity and 90-day mortality following emergency surgery for ALCD.

The elevated risk of mortality in elderly surgical patients is well documented in the literature [5,6,7,8,9,10,11,12,13,14,15,16,17,25]. Duron et al. [10], in a prospective study of 3322 patients undergoing digestive surgery, reported a significant increase in postoperative mortality rates among elderly individuals. Similarly, Lidsky et al. [25], in a large multicenter series, identified age ≥80 years as a significant predictor of postoperative death and also highlighted additional preoperative risk factors including sepsis, malnutrition, chronic steroid use, ASA score ≥ 4, and diabetes mellitus in elderly patients undergoing emergency surgery for diverticulitis.

In contrast, Anegawa et al. [26] did not find a significant difference in postoperative mortality between older and younger patients undergoing emergency surgery for perforated diverticulitis, though they did identify the Mannheim Peritonitis Index (MPI) and ASA score as predictors of mortality.

In our study, a focused subgroup analysis of octogenarians was conducted to identify independent risk factors for postoperative morbidity and mortality. Our findings confirm that chronic renal insufficiency (CRI) and an ASA score ≥ 3 are significant predictors of postoperative morbidity. Moreover, COPD, CRI, ASA score ≥ 3, and MPI > 25 emerged as independent predictors of 90-day postoperative mortality. The MPI, in particular, proved to be a strong predictor of postoperative death. Muralidhar et al. [21], in a prospective study of patients with peritonitis secondary to hollow viscus perforation, used an MPI score of 25 as a cutoff. They reported a mortality rate of 29.4% for MPI ≥ 26 compared to 6.1% for MPI ≤ 25, which was statistically significant (*p* = 0.03). Our study supports this finding: 73.3% of elderly patients with an MPI > 25 died following emergency surgery for ALCD. A limitation of our analysis is the small number of patients in the elderly subgroup, which may affect the stability of the regression model.

The ASA score also remains a valuable preoperative tool to stratify surgical risk. In our series, all elderly patients who died postoperatively had an ASA score ≥ 3, underscoring its prognostic utility. Therefore, our analysis shows that age over 80 years, when combined with an ASA score ≥ 3 and MPI > 25, constitutes an independent risk factor for mortality after emergency surgery for ALCD.

Despite the strong predictive value of these scoring systems, we firmly believe that they should not preclude surgical intervention. Emergency surgery remains the only lifesaving treatment for patients presenting with ALCD.

The main limitation of our study is its retrospective design. However, all data were prospectively collected, all patients undergoing emergency surgery for ALCD during the study period were included, and the entire cohort was analyzed without exclusions.

## 5. Conclusions

Our study demonstrates that advanced age (≥80 years) is an independent risk factor for 90-day postoperative morbidity and mortality following emergency surgery for acute left-sided complicated diverticulitis (ALCD). Among elderly patients, an ASA score ≥ 3, chronic renal insufficiency (CRI), chronic obstructive pulmonary disease (COPD), and a Mannheim Peritonitis Index (MPI) > 25 are significant predictors of poorer prognosis. These findings may assist in preoperative risk stratification and decision-making for elderly patients requiring emergency surgery for ALCD.

## Figures and Tables

**Table 1:** Mannheim Peritonitis Index.

Risk Factor Score	Score
Age > 50 years old	5
Female sex	5
Organ failure *	7
Malignancy	4
Preoperative duration of peritonitis > 24 h	4
Origin of sepsis not colonic	4
Diffuse generalized peritonitis	6
Exudate	
Clear	0
Cloudy, purulent	6
Fecal	12

* Kidney failure, creatinine level > 177 μmol/L or urea level > 167 mmol/L or oliguria < 20 mL/h; pulmonary insufficiency, PO2 < 50 mmHg or PCO2 > 50 mmHg; intestinal obstruction/paralysis > 24 h or complete mechanical ileus, hypodynamic shock, or hyperdynamic.

**Table 2:** Preoperative characteristics of the study population in relation to age.

	Age < 80 Years Group 1: 125 pts.	Age ≥ 80 Years Group 2: 49 pts.	*p*-Value
**Age** (years) Median—Range	67.4 (27–79)	84.1 (80–94)	**0.001**
**Male sex**	72 (57.6%)	16 (32.6%)	**0.001**
**BMI** (Mean ± SD)	24.9 ± 4.6	25.1 ± 4.8	0.799
**Comorbidities**			
*Cardio-Vascular*	57 (45.6%)	44 (89.7%)	**0.001**
*Diabetes*	16 (12.8%)	11 (22.5%)	0.160
*COPD*	20 (16.0%)	13 (26.5%)	0.133
*CRI*	13 (10.4%)	22 (44.9%)	**0.001**
*Immunosuppressed*	13 (10.4%)	3 (6.1%)	0.561
**ASA Classification**			**0.001**
*1*	8 (6.4%)	0	
*2*	45 (36.0%)	1 (2.0%)	
*3*	54 (43.2%)	29 (59.2%)	
*4*	18 (14.4%)	19 (38.8%)	
**Hinchey Classification**			**0.001**
*1b–2*	30 (24.0%)	2 (4.0%)	
*3–4*	85 (76.0%)	47 (96.0%)	
**Mannheim Peritonitis Index**			**0.021**
*≤25*	105 (84.0%)	33 (67.3%)	
*>25*	20 (16.0%)	16 (32.7%)	

COPD, chronic obstructive pulmonary disease. CRI, chronic renal insufficiency.

**Table 3:** Surgical procedures and postoperative outcomes of the study population in relation to age.

	Age < 80 Years Group 1: 125 pts.	Age ≥ 80 Years Group 2: 49 pts.	*p*-Value
**Surgical Procedures**			0.067
*Resection + anastomosis*	26 (20.8%)	3 (6.1%)	
*Resection + ostomy*	90 (72.0%)	44 (89.8%)	
*Laparoscopic Lavage*	9 (7.2%)	2 (4.1%)	
**Duration of procedures (min)**	135.7 ± 38.6	136.6 ± 48.6	0.898
**Clavien-Dindo classification**			**0.001**
*0*	72 (60.1%)	9 (27.3%)	
*1–2*	32 (27.6%)	21 (63.6%)	
*3–4*	12 (10.3%)	3 (9.1%)	
**90-day mortality** (Mean ± SD)	9 (7.2%)	16 (32.6%)	**0.001**
**Hospital stay (days)** (Mean ± SD)	13.5 ± 14.5	14.6 ± 13.1	0.644
**30-day readmission** (Mean ± SD)	8 (6.2%)	4 (8.2%)	0.741

**Table 4:** Factors significantly influencing postoperative morbidity (logistic regression model).

**Parameter**	***p*-Value**	**OR (95% CI)**
Age ≥ 80	0.05	2.6 (1.6–4.8)
Sex	NS	-
Cardio-Vascular comorbidities	0.01	2.8 (1.9–3.6)
CRI	0.01	5.1 (2.1–7.4)
ASA score	0.03	2.5 (2.0–4.8)
Hinchey 3–4	NS	-
Mannheim Peritonitis Index > 25	0.02	2.9 (1.5–4.2)

CRI, chronic renal insufficiency; NS, not significant.

**Table 5:** Factors significantly influencing 90-day mortality (logistic regression model).

Parameter	*p*-Value	OR (95% CI)
Age ≥ 80	0.08	1.8 (1.1–2.5)
Sex	NS	-
Cardio-Vascular comorbidities	NS	-
CRI	0,22	3.9 (1.9–5.6)
ASA score	0.01	6.3 (2.6–8.8)
Hinchey 3–4	NS	-
Mannheim Peritonitis Index > 25	0,01	3.4 (2.4–6.4)

CRI, chronic renal insufficiency; NS, not significant.

**Table 6:** Factors associated with postoperative morbidity in octogenarians.

	NO Postoperative Morbidity (n = 9)	YES Postoperative Morbidity (n = 40)	*p*-Value
**Male sex**	5 (55.5%)	11 (27.5%)	0.130
**BMI** (Mean ± SD)	24.8 ± 4.7	25.1 ± 4.9	0.868
**Comorbidities**			
*Cardio-Vascular*	5 (55.5%)	39 (97.5 %)	**0.027**
*Diabetes*	0	11 (27.5%)	0.098
*COPD*	2 (22.2%)	11 (27.5%)	1.000
*CRI*	0	22 (55.0%)	**0.025**
*Immunosuppressed*	1 (11.1%)	2 (5.0%)	0.463
**ASA Classification**			**0.049**
*1*	0	0	
*2*	1 (11.1%)	0	
*3*	7 (77.8%)	22(55.0%)	
*4*	1 (11.1%)	18 (45.0%)	
**Hinchey Classification**			0.337
*1b–2*	1 (11.1%)	1 (2.5%)	
*3–4*	8 (88.9%)	39 (97.5%)	
**Mannheim Peritonitis Index**			0.238
*≤25*	8 (88.8%)	25 (62.5%)	
*>25*	1 (11.2%)	15 (37.5%)	
**Surgical procedures**			0.571
*Resection + anastomosis*	1 (11.1%)	2 (5.0%)	
*Resection + ostomy*	8 (88.9%)	36 (90.0%)	
*Laparoscopic Lavage*	0	2 (5.0%)	
**Duration of procedures (min)**			0.755
Mean ± SD	128.8 ± 27	132.4 ± 32	

COPD, chronic obstructive pulmonary disease. CRI, chronic renal insufficiency.

**Table 7:** Factors associated with 90-day mortality in octogenarians.

	NO 90-Day Mortality (n = 33)	YES 90-Day Mortality (n = 16)	*p*-Value
**Male sex**	8 (24.2%)	8 (50.0%)	0.106
**BMI** (Mean ± SD)	25.1 ± 4.5	24.8 ± 4.8	0.831
**Comorbidities**			
*Cardio-Vascular*	28 (84.8%)	16 (100 %)	0.158
*Diabetes*	5 (9.1%)	6 (37.5%)	0.141
*COPD*	5 (9.1%)	8 (50.0%)	**0.016**
*CRI*	11 (33.3%)	11 (68.7%)	**0.031**
*Immunosuppressed*	1 (3.3%)	2 (12.5%)	0.245
**ASA Classification**			**0.004**
*1*	0	0	
*2*	1 (3.2%)	0	
*3*	24 (74.2%)	5 (26.7%)	
*4*	7 (22.6%)	12 (73.3%)	
**Hinchey Classification**			1.000
*1b–2*	2 (6.1%)	0	
*3–4*	31 (93.9%)	16(100%)	
**Mannheim Peritonitis Index**			**<0.001**
*≤25*	28 (84.8%)	5 (31.2%)	
*>25*	5 (15.2%)	11 (68.8%)	
**Surgical procedures**			0.347
*Resection + anastomosis*	2 (6.1%)	1 (2.3%)	
*Resection + ostomy*	29 (87.8%)	15 (93.7%)	
*Laparoscopic Lavage*	2 (6.1%)	0	
**Duration of procedures (min)**			0.923
Mean ± SD	136.7 ± 46	135.4 ± 38	

COPD, chronic obstructive pulmonary disease. CRI, chronic renal insufficiency.

**Table 8:** Factors significantly influencing postoperative morbidity and 90-day mortality in octogenarians (logistic regression model).

Parameter	*p*-Value	OR (95% CI)
**Postoperative Morbidity**		
Cardio-Vascular comorbidities	NS	-
CRI	0.02	2.9 (1.9–3.6)
ASA score	0.03	4.1 (1.4–10.3)
**90-Day Mortality**		
COPD	0.04	6.2 (1.1–35.9)
CRI	0.02	10.2 (1.4–69.2)
ASA score	0.02	6.5 (1.1–32.9)
Mannheim Peritonitis Index > 25	0.02	3.5 (1.3–7.3)

COPD, chronic obstructive pulmonary disease. CRI, chronic renal insufficiency.

## Data Availability

The datasets generated and analysed for this study are not publicly available, as they were collected by different individuals from multiple organisations with varying data-sharing policies. Requests for access should be directed to the corresponding author, who may seek permission to share specific anonymized data from all relevant parties.

## Abbreviations

The following abbreviations are used in this manuscript:

ALCD	acute left-sided complicated diverticulitis
COPD	chronic obstructive pulmonary disease
CRI	chronic renal insufficiency
MPI	Mannheim Peritonitis Index
